# The Nutritional Induction of COUP-TFII Gene Expression in Ventromedial Hypothalamic Neurons Is Mediated by the Melanocortin Pathway

**DOI:** 10.1371/journal.pone.0013464

**Published:** 2010-10-18

**Authors:** Lina Sabra-Makke, Cécile Tourrel-Cuzin, Raphaël G. P. Denis, Marthe Moldes, Jean-Paul Pégorier, Serge Luquet, Mireille Vasseur-Cognet, Pascale Bossard

**Affiliations:** 1 Institut Cochin, Université Paris Descartes, Department of Endocrinology, Metabolism and Cancer, CNRS (UMR 8104), Paris, France; 2 INSERM, U1016, Paris, France; 3 Laboratoire de Biologie et Pathologie du Pancréas Endocrine, Unité “Biologie Fonctionnelle & Adaptative”, Université Paris Diderot-Paris 7, CNRS EAC 4413, Paris, France; 4 Team HERGE, Unité “Functional and Adaptive Biology, Biologie Fonctionnelle & Adaptative”, Université Paris Diderot-Paris 7, CNRS EAC 4413, Paris, France; INSERM, France

## Abstract

**Background:**

The nuclear receptor chicken ovalbumin upstream promoter transcription factor II (COUP-TFII) is an important coordinator of glucose homeostasis. We report, for the first time, a unique differential regulation of its expression by the nutritional status in the mouse hypothalamus compared to peripheral tissues.

**Methodology/Principal Findings:**

Using hyperinsulinemic-euglycemic clamps and insulinopenic mice, we show that insulin upregulates its expression in the hypothalamus. Immunofluorescence studies demonstrate that COUP-TFII gene expression is restricted to a subpopulation of ventromedial hypothalamic neurons expressing the melanocortin receptor. In GT1-7 hypothalamic cells, the MC4-R agonist MTII leads to a dose dependant increase of COUP-TFII gene expression secondarily to a local increase in cAMP concentrations. Transfection experiments, using a COUP-TFII promoter containing a functional cAMP responsive element, suggest a direct transcriptional activation by cAMP. Finally, we show that the fed state or intracerebroventricular injections of MTII in mice induce an increased hypothalamic COUP-TFII expression associated with a decreased hepatic and pancreatic COUP-TFII expression.

**Conclusions/Significance:**

These observations strongly suggest that hypothalamic COUP-TFII gene expression could be a central integrator of insulin and melanocortin signaling pathway within the ventromedial hypothalamus. COUP-TFII could play a crucial role in brain integration of circulating signal of hunger and satiety involved in energy balance regulation.

## Introduction

The hypothalamus receives information from circulating nutrients and hormones and integration of these signals by appropriate neurocircuitry will in turn translate into adaptive metabolic and behavioral responses in order to sustain energy balance. Food intake modulation, insulin secretion and peripheral glucose metabolism together with endocrine regulation are part of this integrated response. Any disturbance of this mechanism leads to metabolic diseases such as obesity and diabetes [1], [2].

Nuclear receptors are part of a family of ligand-activated transcription factor such as PPARs, LXRs, RXR, involved in the regulation of many key metabolic functions both at the central and peripheral level. Among these transcription factors, our team has focused its attention on the Chicken Ovalbumin Upstream Promoter-Transcription Factor II (COUP-TFII also named NR2F2). COUP-TFII is an orphan member of the steroid/thyroid hormone receptor superfamily with no identified physiological ligand. It binds DNA by a zinc finger motif on a variety of hormone responsive elements that contain direct or inverted imperfect AGGTCA repeats with various spacings [3]. COUP-TFII is involved in the control of development, cellular differentiation, growth and metabolism [4], [5], [6]. In terms of metabolic control, we and others showed its functional role in respect to genetic programs associated with insulin biosynthesis and secretion in pancreatic beta cells [7], [8], in the regulation of lipid utilization/cholesterol homeostasis in skeletal muscle cells [9] and in white adipose tissue development and energy metabolism [10]. The fact that heterozygous COUP-TFII gene inactivation mouse models, *i.e* conditional beta cells [8] or complete invalidation [10] (50% decrease in COUP-TFII expression) displayed a strong phenotype suggests that any small variations of COUP-TFII expression does lead to a disturbed physiology.

Interestingly, its expression is modulated by the nutritional status in several tissues. We previously reported that COUP-TFII expression was reduced in the pancreas and liver of mice refed onto with a carbohydrate rich diet [7]. In fact, COUP-TFII gene expression is decreased by secreted insulin in response to glucose in pancreatic beta cells and by insulin and glucose in hepatocytes [7]. *In situ* hybridization data showed that COUP-TFII mRNA was also expressed in the hypothalamus [11], [12].

As an actor of glucose homeostasis, whose expression is modulated by the nutritional status in several tissues and as it is expressed in the hypothalamus whose function is central to energy homeostasis, it was of interest to decipher the regulation of COUP-TFII expression by the nutritional status in the hypothalamus.

In this paper, we report 1) a differential regulation of COUP-TFII mRNA levels by the nutritional status with an induction of its expression in the hypothalamus in the fed state 2) COUP-TFII protein expression in a subpopulation of ventromedial hypothalamic neurons 3) COUP-TFII expression in the hypothalamus and in a hypothalamic cell line being controlled by the melanocortin pathway leading to a direct COUP-TFII transcriptional activation.

## Results

### Hypothalamic COUP-TFII expression is induced by the fed state in adult mice

**Figure 1:**
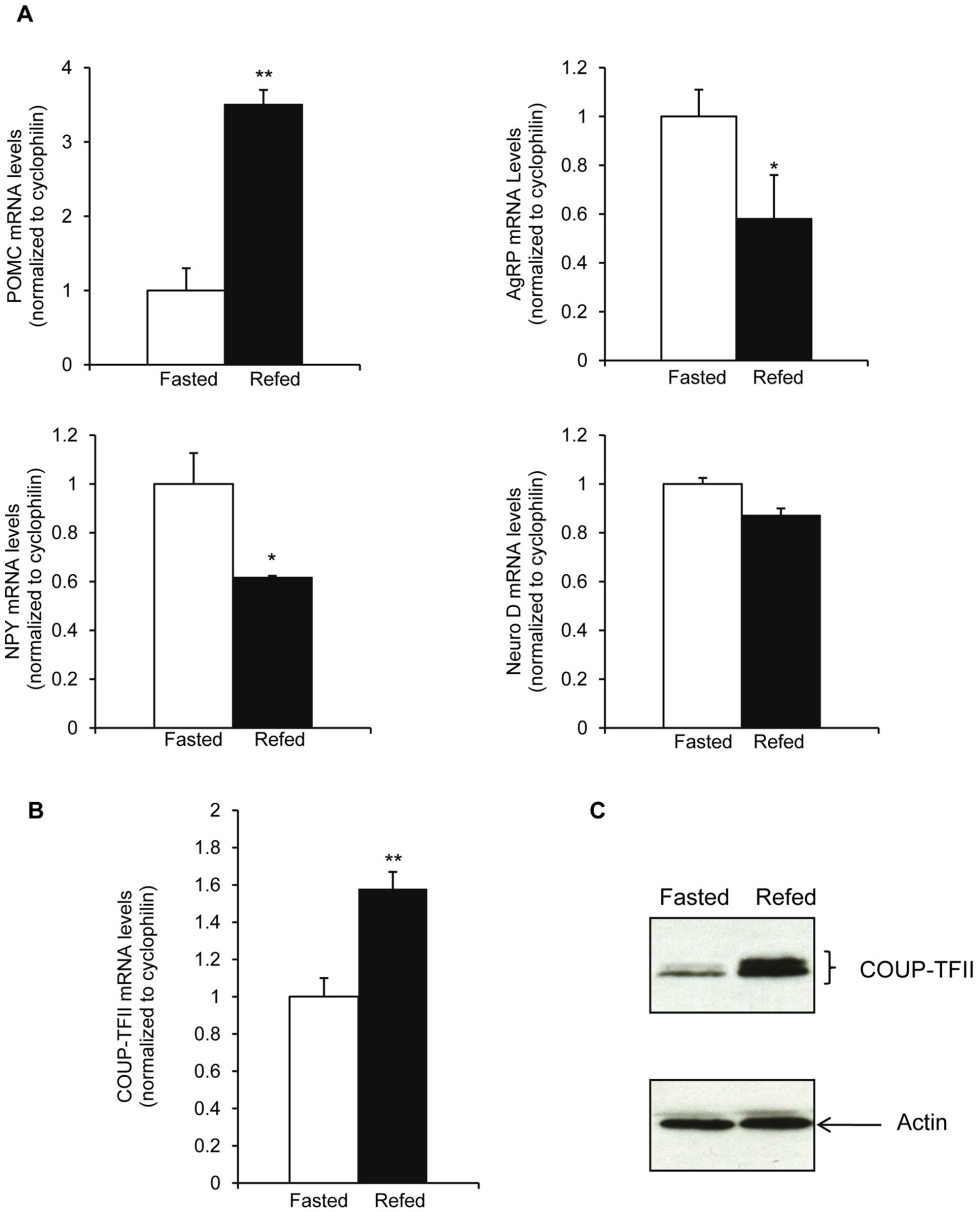
In vivo COUP-TFII expression in the hypothalamus during the fasting refeeding cycle. C57BL/6J mice are either fasted for 24 h (□) or fasted 24 h and refed on a regular diet (▪). (A) RT-qPCR analysis of POMC (upper left panel), AgRP mRNA levels (upper right panel), NPY mRNA (lower left panel), Neuro D mRNA levels (lower right panel) (B) RT-qPCR analysis of COUP-TFII mRNA levels. Data are expressed as mean ± SEM. (*n* = 5 mice/group) *P≤0.05; **P≤0.01 when compared to fasted conditions. (C) Autoradiogram of a western blot analysis of COUP-TFII protein expression in the hypothalamus. Upper panel: COUP-TFII protein, lower panel: β actin protein.

Previous studies from our lab and from others had observed an expression of COUP-TFII mRNA in embryonic and adult hypothalamus [12], [13], [14]. From our previous studies on COUP-TFII mRNA regulation in liver and pancreas, we looked at the regulation of COUP-TFII mRNA levels in the hypothalamus of C57bl/6J adult mice according to their nutritional status. Mice were subjected to a 24 hours fast and compared with mice refed a regular chow diet for 6 hours, which causes a major switch leading to elevated plasma glucose (from 102±10.2 mg/dl at the fasted state to 165±9.5 mg/dl in the fed mice) and plasma insulin levels (from 0.6±0.2 at the fasted state to 1.9±0.3 in the fed mice). In the hypothalamus, the fed state is characterized by increased proopiomelanocortin (POMC) and decreased agouti related protein (AgRP) mRNA and neuropeptide Y (NPY) mRNA contents [15], [16], [17], as observed in our mice (figure 1A). Refed mice displayed significantly higher COUP-TFII mRNA levels (48%) in the hypothalamus than the fasted mice (figure 1B). In these experiments, we analyzed also COUP-TFII protein expression. We observed a significantly higher expression in the hypothalamus of the fed mice than in the hypothalamus of the fasted mice with the characteristic migration profile with 2 bands, the minor higher molecular weight band corresponding to a post translational modified COUP-TFII protein [18]. In these experiments, the ratio of the two bands was modified in a similar way between the fasted and the fed state (figure 1C). Taken altogether, these results suggested that the changes in hypothalamic COUP-TFII expression could be centrally regulated by signals of energy supplies such as blood glucose or plasma insulin levels. To confirm this hypothesis, we investigated the effects of glucose and/or insulin in the control of hypothalamic COUP-TFII mRNA levels.

**Figure 2:**
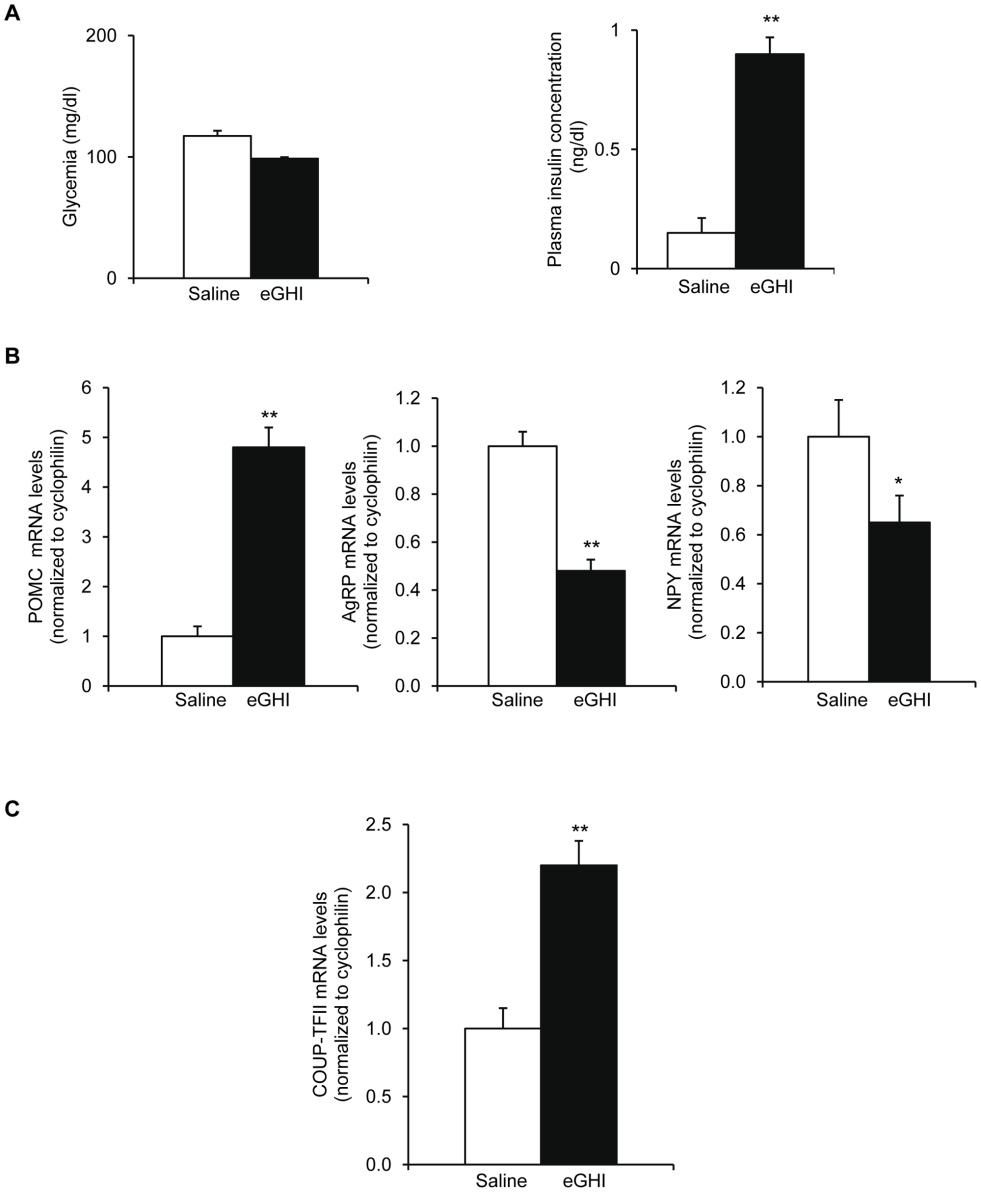
COUP-TFII mRNA levels in the hypothalamus of eGHI clamped mice. (A) Blood glucose and plasma insulin (B-C) RT-qPCR analysis of mRNA levels in the hypothalamus of eGHI clamped mice. (B) Left panel: POMC mRNA levels; middle panel: AgRP mRNA levels; right panel: NPY mRNA levels (C) COUP-TFII mRNA levels. Saline control mice (□), eGHI (▪) Data are expressed as mean ± S.E.M. (*n* = 6 mice/group). **P≤0.01 when compared to saline control mice.

As insulin and glycemia are so tightly linked, in order to distinguish between the relative implication of glucose or insulin on hypothalamic COUP-TFII regulation, we used the euglycemic/hyperinsulinemic (eGHI) clamp technique to impose high circulating insulin while maintaining euglycemia with an intra-jugular glucose infusion (figure 2A). First, we analyzed POMC, AgRP and NPY mRNAs levels in these clamped mice (figure 2B) and observed a significant 3 fold increase in POMC mRNA levels and a 2.3 and 1.6 fold decrease respectively in AgRP and NPY mRNA levels in the eGHI mice compared to the control mice (figure 2B). In these experiments, COUP-TFII mRNA levels were significantly increased by a 2.2 fold in the eGHI clamped mice compared to the control mice (figure 2C). Thus, insulin *per se* was able to induce COUP-TFII mRNA levels in the hypothalamus independently of glucose.

**Figure 3:**
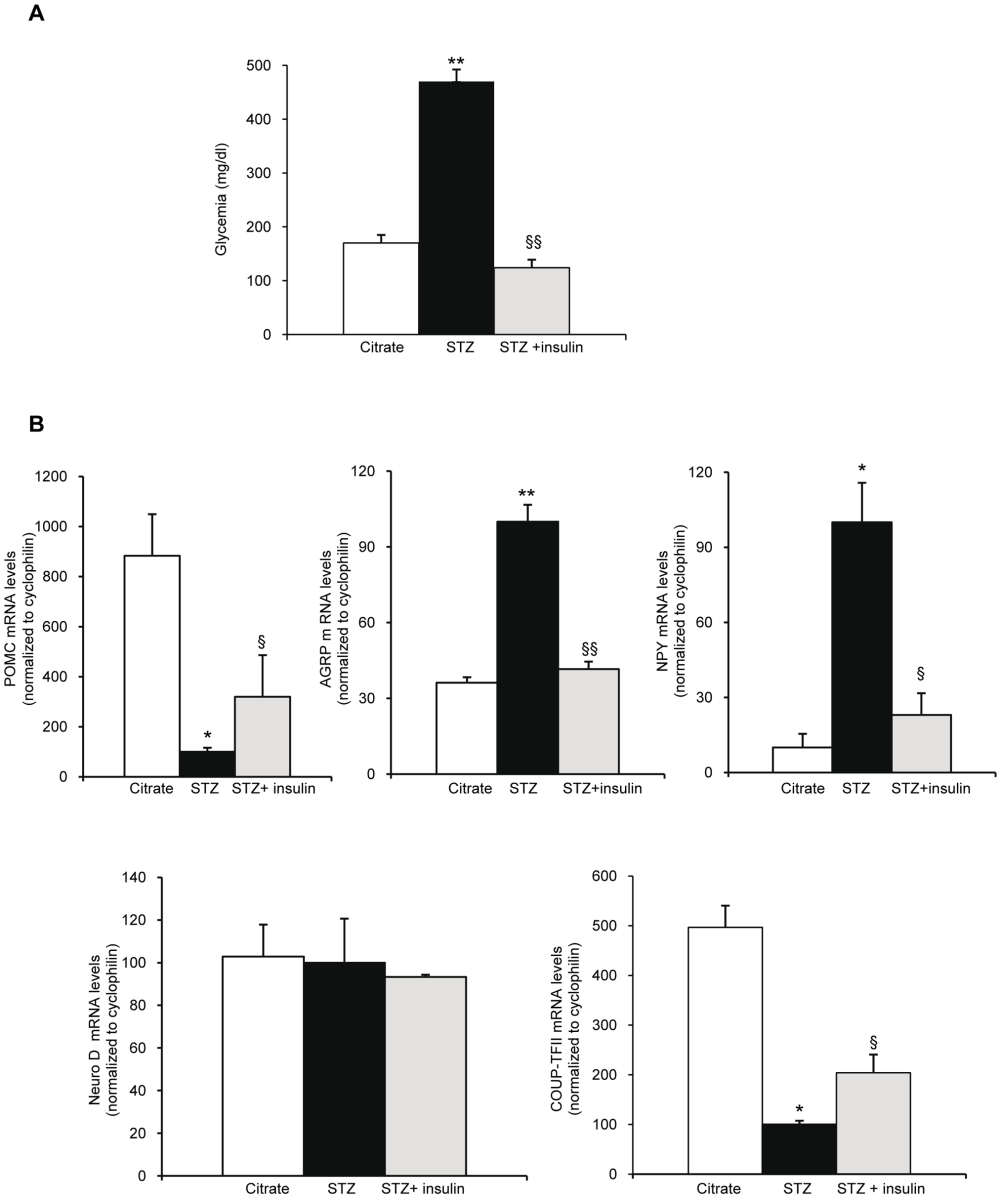
COUP-TFII mRNA levels in the hypothalamus of STZ treated mice. (A) Glycemia (B) RT-qPCR analysis of mRNA levels. Upper left panel: POMC mRNA levels; upper middle panel: AgRP mRNA levels; upper right panel: NPY mRNA levels; lower left panel: Neuro-D mRNA levels; lower right panel: COUP-TFII mRNA levels. Citrate control mice (□), STZ treated mice (▪), STZ mice treated with insulin (grey square). Data are expressed as mean ± SEM. (*n* = 6 mice/group). *P≤0.05 **P≤0.01 compared to citrate treated mice. § P≤0.05 §§P≤0.01 compared to STZ treated mice.

Conversely, we analyzed COUP-TFII mRNA levels in the hypothalamus of insulinopenic mice. We generated type 1 diabetic mouse models using streptozotocin injections. The control mice received citrate buffer injections (the streptozotocin vehicle). Once the hyperglycemia (470±50 mg/dl) was stable (*i.e*. 8 days) in the STZ mice, the hypothalamic gene expression was analyzed (figure 3A). In parallel, a subgroup of STZ treated mice received an insulin injection (1.5 U/kg) two hours prior to the sacrifice. At the time of sacrifice, the glycemia of these insulin injected STZ mice had dropped down to 120±27 mg/dl (figure 3A). In the STZ treated mice, POMC mRNA levels were drastically decreased by a 8 fold, whereas in the STZ subgroup receiving the insulin injection, POMC mRNA levels were reinduced by a 3.4 fold. Conversely, hypothalamic AgRP and NPY mRNA levels were respectively 3 and 10 fold higher in STZ treated mice than in the control citrate mice, however the insulin injection rapidly repressed their expressions (figure 3B). In these experiments, COUP-TFII mRNA levels dropped dramatically (80%) in insulinopenic mice compared to their control mate whereas exogenous insulin injection induced a rapid and significant increase in COUP-TFII mRNA levels within 2 hours compared to the STZ mice (figure 3B). Taken all together, these results showed that insulin is able to induce a rapid increase in hypothalamus COUP-TFII gene expression. These results are quite intriguing since our previous data had clearly demonstrated that COUP-TFII gene expression was decreased by insulin in hepatocytes and in pancreatic β cells and decreased in liver and pancreas of fed mice [7]. Therefore, we questioned how the fed state could have such an opposite effect on COUP-TFII mRNA expression in the hypothalamus compared to the liver and the pancreas.

### COUP-TFII expression is restricted to a subpopulation of VMH neurons

**Figure 4:**
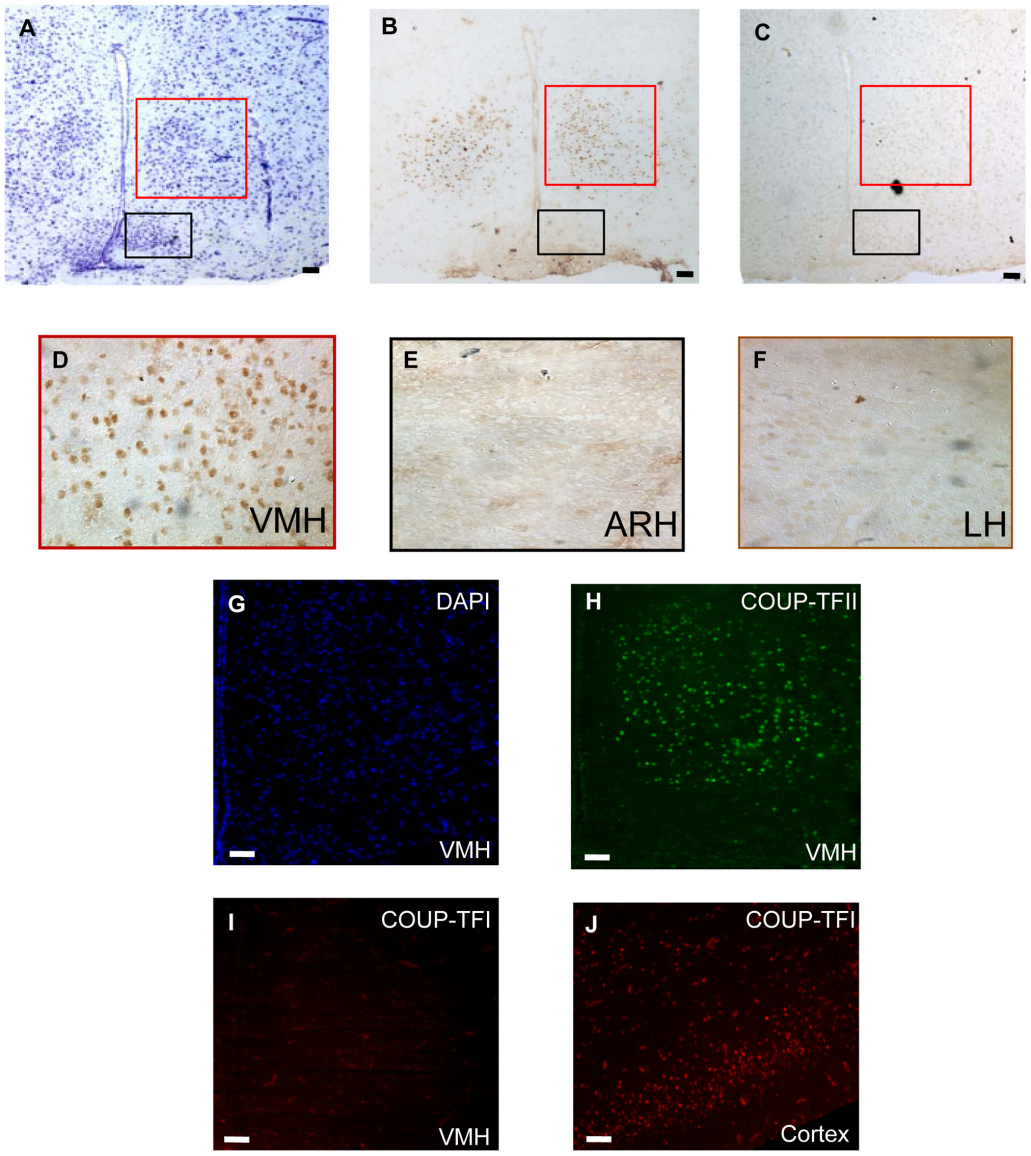
COUP-TFII protein expression in adult mouse ventromedial hypothalamus. (A) Identification of the hypothalamic structures by Kluver Barrera staining. Black square: Arcuate nucleus, red square: Ventromedial hypothalamic nucleus. (B–F) Immunostaining of brain sections from adult mice using mouse monoclonal antibodies directed against COUP-TFII (PP-H7147-00, dilution 1∶50). The secondary antibody is coupled with the horse radish peroxydase and revealed with diaminobenzidine leading to a brown precipitate. The sections shown are from the same experiment with the same exposure times. Magnification ×20. (C) Secondary antibody incubation control for COUP-TFII antibody specificity (D) Enlargement from the VMH region, magnification ×40. (E) Enlargement of the arcuate nucleus region (ARH), magnification ×40. (F) Enlargement from the LHA region, magnification ×40. These pictures are representative of 6 independent experiments. (G–J) Immunofluorescence staining of adult mice hypothalamic cryostat sections, magnification ×20. (G) DAPI staining of the ARH-VMH region (blue fluorescence) (H) Identification of COUP-TFII positive neurons in the VMH-ARH region of adult mice using the COUP-TFII monoclonal antibody (green fluorescence) (I) Identification of COUP-TFI positive neurons in the VMH-ARH region of the hypothalamus using a COUP-TFI monoclonal antibody (red fluorescence) (J) Identification of COUP-TFI positive neurons in the cortex region of the same cryostat section than (I) as a positive control for COUP-TFI antibody. Scale bars  = 100 µm.

The hypothalamus is a complex structure consisting of anatomically distinct nuclei that work in a coordinate fashion to regulate vegetative functions [19]. In order to decipher the mechanisms by which insulin is able to stimulate hypothalamic COUP-TFII gene expression, we needed to identify precisely the nuclei expressing this transcription factor. Therefore, we did a study of COUP-TFII protein expression profile by immunohistochemistry on cryostat sections of C57bl/6J fed mice brains (figure 4). After a Kluver Barrera staining, the hypothalamic nuclei were identified using a mouse brain atlas as well as morphological brain markers (figure 4A). Throughout all hypothalamic sections, we detected the COUP-TFII protein only in the ventromedial nuclei (VMH) with the very characteristic perinuclear profile (figure 4B–H). This protein expression profile is reminiscent of the COUP-TFII mRNA expression profiles observed by *in situ* hybridization studies (Allen Brain Atlas). These data confirmed the highly restricted expression of the protein. The secondary antibody specificity was confirmed on cryostat sections done without primary antibody incubation (figure 4C). Similar experiments using a primary monoclonal antibody directed against the COUP-TFI homologue, a closely related protein, were done and no expression was observed in the VMH while it was clearly expressed in cortical subpopulations on the same cryostat section, as previously described [20] (figure 4I–J).

**Figure 5:**
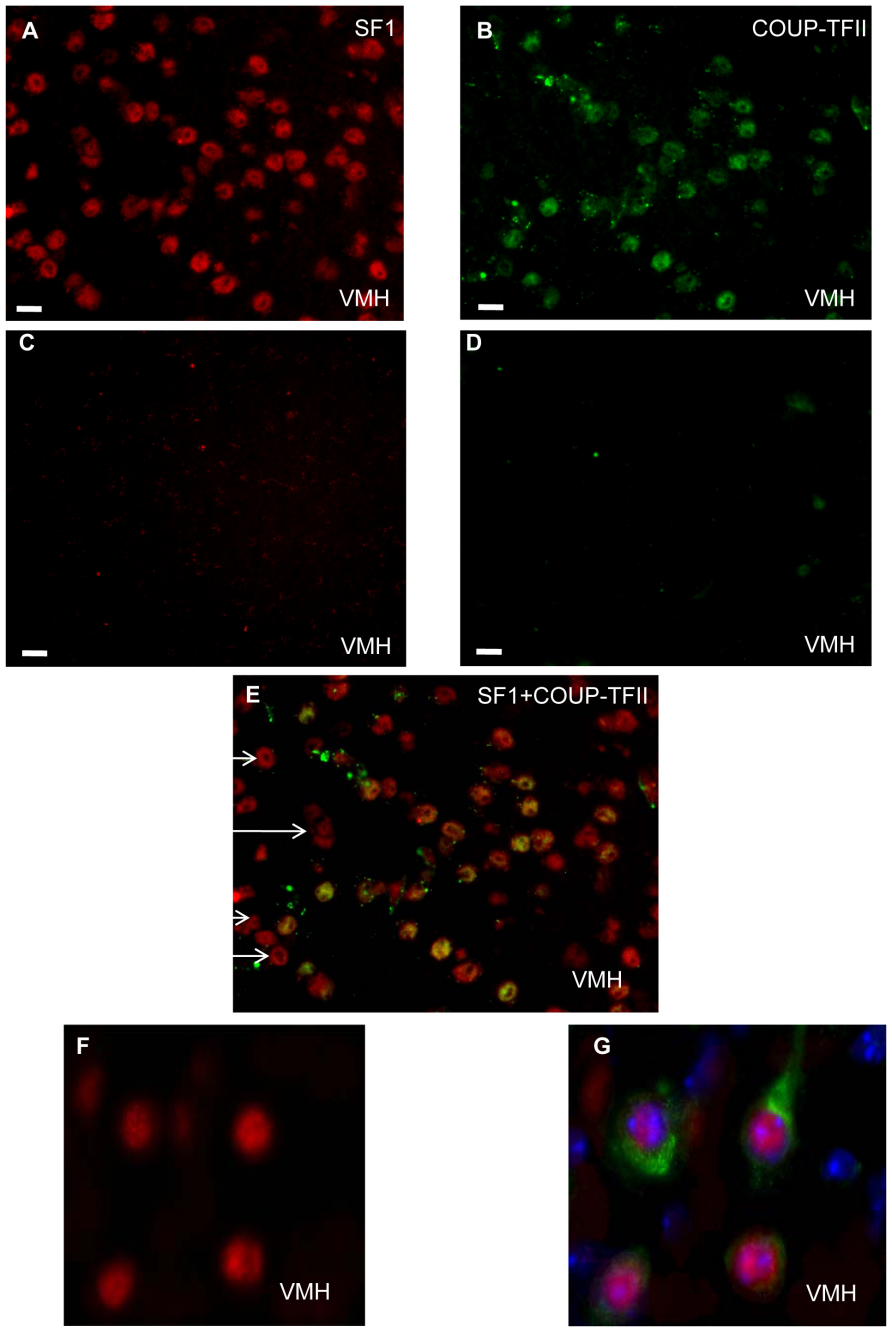
COUP-TFII protein co-localization studies with SF1 in the VMH. (A–G) Immunofluorescence staining of adult mice hypothalamic cryostat sections. (A–E) The sections shown are from the same experiment with the same exposure time (A) Identification of SF1 positive cells in the VMH by immunofluorescence using a SF1 polyclonal antibody (red fluorescence) (B) Identification of the COUP-TFII positive neurons in the VMH of adult mice using a COUP-TFII monoclonal antibody (green fluorescence) (C) Secondary antibody alone control for SF1 antibody specificity (D) Secondary antibody alone for COUP-TFII antibody specificity (E) Superimposed SF1 and COUP-TFII immunostaining. Magnification ×40. (F–G) The sections shown are from the same experiment and the same exposure time. (F) COUP-TFII immunostaining (red fluorescence) (G) Superimposed MC4-R and COUP-TFII immunofluorescences. Magnification ×100. These pictures are representative of 4 independent experiments. Scale bars  = 10 µm.

The VMH remains poorly defined in terms of cellular and molecular genetic markers as tool to identify cell subgroups. Recently a transcriptome analysis of the VMH has allowed the identification of some specific markers of the different cell subpopulations [12]. Among them, COUP-TFII had one of the highest levels of expression [12]. In order to delineate if COUP-TFII was more specifically expressed in neurons or astrocytes, we undertook a co-localization study with an astrocyte specific marker, the Glial Fibrilary Acidic Protein (GFAP). No overlapping expression with COUP-TFII protein was observed, suggesting that COUP-TFII was not expressed in astrocytes (data not shown). Among the identified VMH markers, the nuclear receptor steroidogenic factor 1 (SF1) is expressed in a large subpopulation of neurons involved in the central control of glucose homeostasis [21], [22]. We addressed the possible overlap between COUP-TFII and SF1 expression within the VMH with a double immunostaining for both proteins. A large number of neurons expressing the SF1 protein were observed in the VMH (figure 5A, red fluorescence). Interestingly, not all VMH cells were expressing COUP-TFII (figure 5B, green fluorescence). By overlapping the two fluorescences, we observed that all the COUP-TFII positive neurons were also expressing the SF1 protein (overlapping green and red fluorescence) (figure 5E, yellow fluorescence). However, some SF1 cells were not expressing COUP-TFII (white arrows), suggesting that we had at least two distinct cellular subpopulations of SF1 neurons within the VMH, the COUP-TFII positive and the COUP-TFII negative neurons with 65% of the SF1 positive cells expressing COUP-TFII (figure 5E). On the other hand, the COUP-TFII neurons were all SF1 expressing neurons (figure 5E, yellow fluorescence). Even so it was known that COUP-TFII mRNA and SF1 mRNA were present in the VMH, this study allowed the first identification of a subpopulation among the VMH neurons expressing both the COUP-TFII and SF1 proteins.

The SF1 positive neurons have been characterized previously as part of the circuitry controlling energy homeostasis and reproductive behavior and are known to express the melanocortin receptor 4 (MC4-R) [22], [23], [24], [25]. We confirmed the presence of this receptor in the COUP-TFII positive neurons by co-localization experiments and found that COUP-TFII neurons (figure 5F, red fluorescence) are indeed MC4-R positive neurons (green fluorescence) (figure 5G), confirming the presence of MC4-R on SF1 neurons. To summarize, COUP-TFII is expressed in the VMH in the SF1 positive neurons, part of the melanocortin pathway.

### COUP-TFII gene expression is modulated by the melanocortin pathway in the GT1-7 hypothalamic cell line

In the central nervous system, the fed state is characterized by the production of α melanocyte-stimulating hormone (αMSH), the hypothalamic POMC product which is synthesized by the neurons of the ARH in response to insulin [26]. The αMSH receptors, MC4-R and MC3-R, are present on numerous neurons from the different hypothalamic nuclei, including the lateral hypothalamus (LH) the paraventricular hypothalamus (PVN) and the VMH where the COUP-TFII neurons are located (figure 5) [27], [28]. COUP-TFII being expressed on neurons known to express the MC4-R, we then checked whether an activation of this receptor could contribute to COUP-TFII expression.

**Figure 6:**
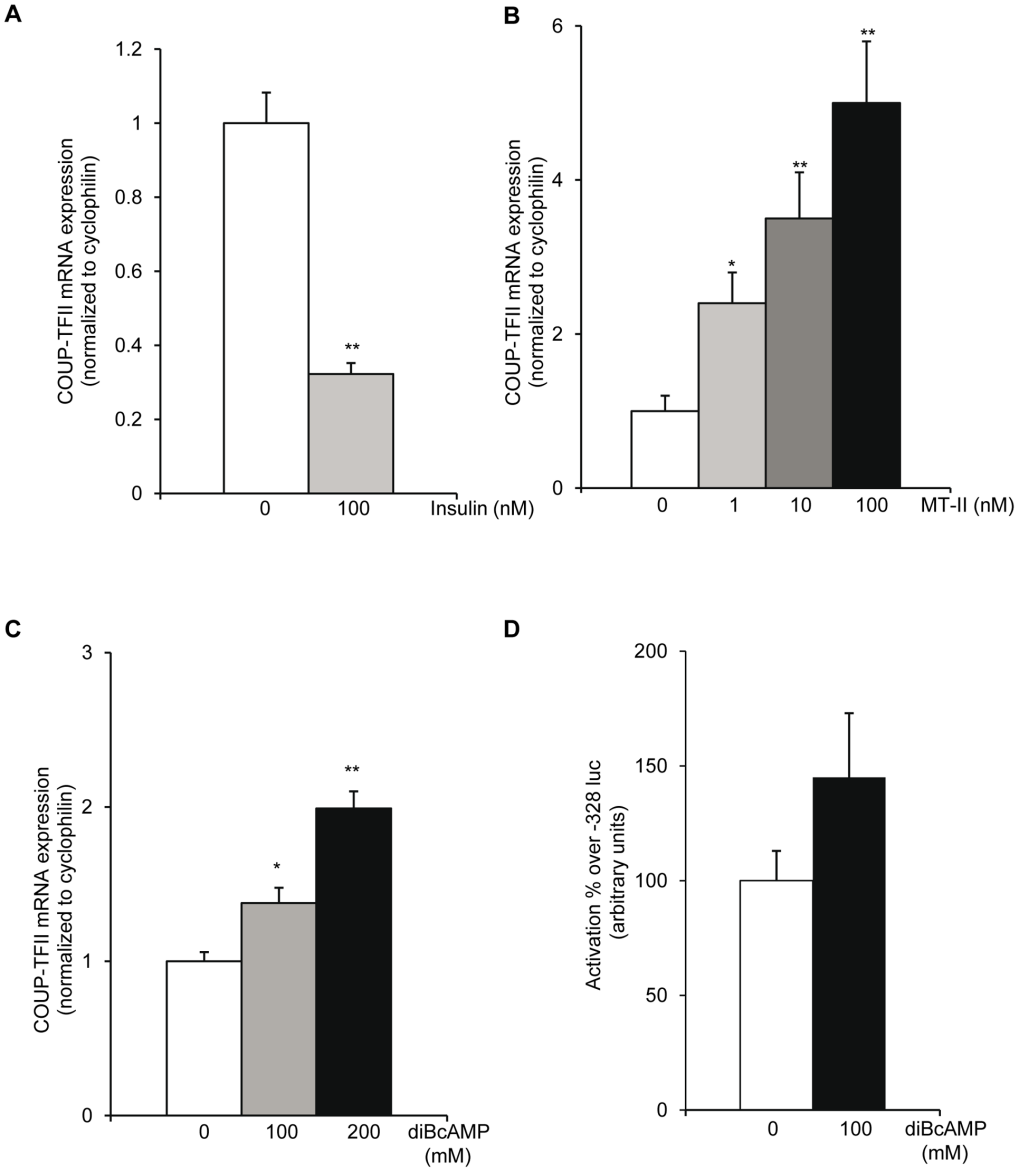
COUP-TFII gene expression in response to the MC4-R activation in GT1-7 cell line. GT1-7 cells were cultured for 18 hours in the presence of either (A) insulin (100 nM) or increasing amount of (B) MTII or (C) DiBcAMP. Results are expressed as a fold increase over the value obtained without MTII (A) or without DiBcAMP (B). (D) Transient transfection of a minimal COUP-TFII promoter in response to DiBcAMP. Data are expressed as the mean ± SEM. (*n* = 4). *P≤0.05 **P≤0.01 when compared to cells cultured in the absence of MTII or DiBcAMP.

We therefore investigated the effect of insulin or of a MC3/4-R agonist on hypothalamic COUP-TFII gene expression in a hypothalamic mouse cell line, the GT1-7 cells, known to express the insulin receptor, the COUP-TFII protein (data not shown) as well as the MC4-R receptor [29].First, the cells were cultured for 18 hours with 100 nM insulin. Under these conditions, we observed a strong repression of COUP-TFII mRNA levels by insulin in cells directly exposed to insulin (figure 6A). On the other hand, when the cells are cultured for 18 hoursin the presence of increasing amounts of the MC4-R agonist, the melanotan II (MTII), a αMSH analog, a concentration of 1 nM MTII is sufficient to cause a significant increase of COUP-TFII mRNA levels in the dose-response study (figure 6B). The MC4-R is a Gα s-coupled receptor known to stimulate cAMP production following agonist stimulation [30]. Indeed, we observed a dose dependant accumulation of COUP-TFII mRNA in response to increasing amount of the cAMP analog DiB-cAMP (figure 6C).

Previous studies from Soosaar *et al*. [31] had identified a functional cAMP response element (CRE) binding site in the promoter region of the mouse COUP-TFII gene. Following this observation, we asked whether cAMP had a direct transcriptional effect on the COUP-TFII gene promoter in an hypothalamic cell line. To demonstrate the direct implication of cAMP in the control of the COUP-TFII gene promoter, the GT1-7 cells were transiently transfected with a reporter plasmid carrying the luciferase gene controlled by the 328 bp 5′ regulatory region. We observed that the cAMP treatment induces in these neuronal cells the -328/luc COUP-TFII construct (figure 6D).

All the experiments performed in this hypothalamic cell line showed that COUP-TFII gene expression was increased by an activation of the MC4-R signaling pathway. This led us to hypothesize that, *in vivo*, the fed state induced COUP-TFII expression through a local activation of the melanocortin pathway in the VMH.

### *In vivo* MC3/4-R activation induces COUP-TFII mRNA levels in the hypothalamus

Therefore we tested the effects of intracerebroventricular (ICV) injections of the MC3/4-R agonist MTII on COUP-TFII mRNA levels in the hypothalamus. In our ICV experiments, mice were subdivided into three groups: one group fed *ad libitum* on a regular laboratory chow received an ICV injection of cerebrospinal fluid (CSF). A second group fasted for 18 hours prior to the experiment received an ICV injection of CSF, the last group fasted for 18 hours received a single MTII injection. Mice were sacrificed 4 hours after the injections.

**Figure 7:**
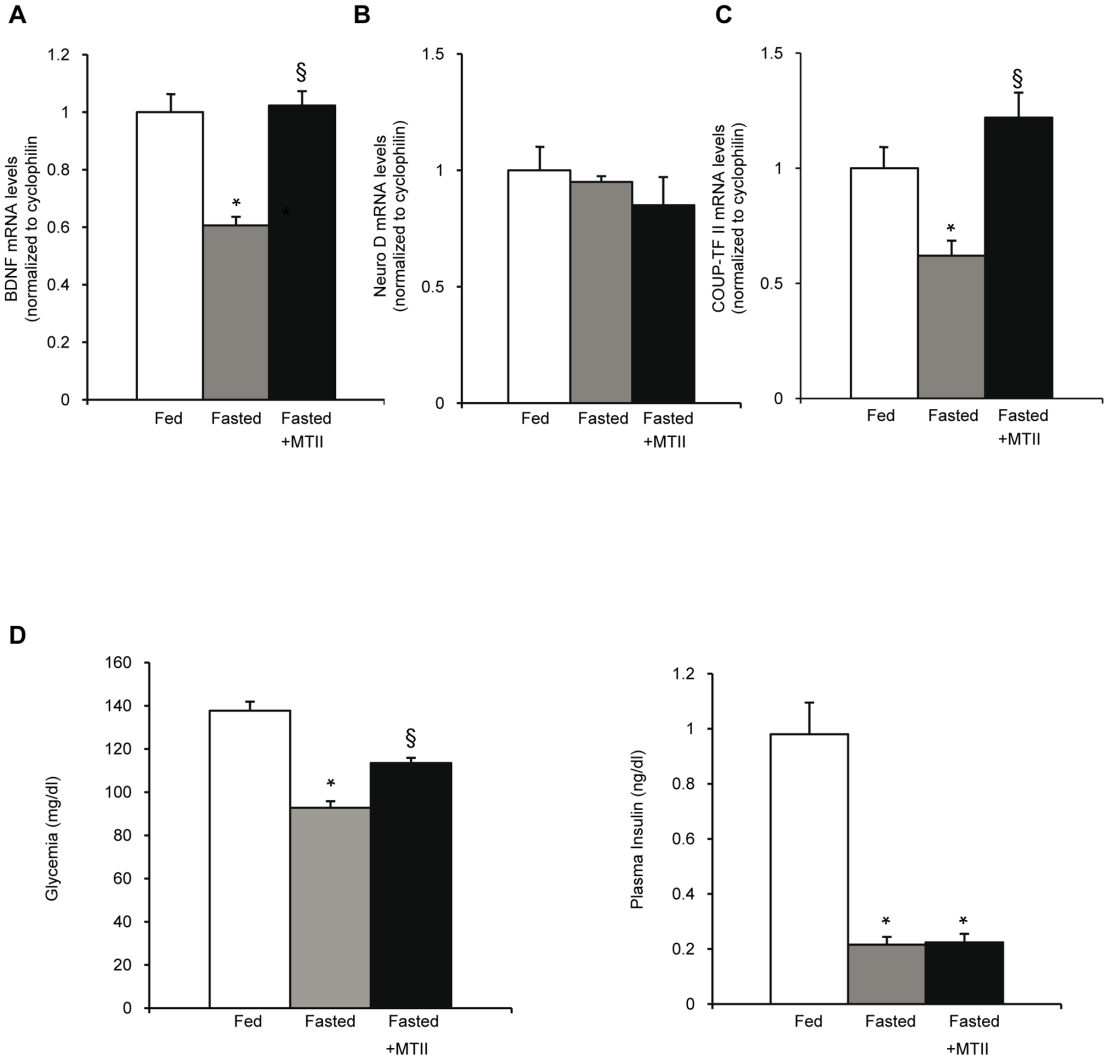
Regulation of hypothalamic COUP-TFII mRNA levels in vivo by activation of the melanocortin pathway. (A-C) RT-qPCR analysis of mRNA levels from hypothalamus of *ad libitum* fed mice receiving a ICV injection of CSF (□), or 18 h fasting mice receiving a ICV injection of CSF (grey bar), or 18 h fasting mice with a ICV injection of MTII (▪). (A) BDNF mRNA levels (B) Neuro D mRNA levels (C) COUP-TFII mRNA levels. Data are expressed as mean ± SEM (n = 5) *P≤0.05 **P ≤0.01 when compared to control fed mice receiving a CSF injection. § P≤0.05 when compared to control fasting mice receiving a CSF injection. (D) Glucose and plasma insulin concentrations from the different mouse models.

As an activation of the hypothalamic MC3/4-Rs is known to induce the expression of the brain derived neurotrophic factor (BDNF) expression, a neurotrophin involved in energy homeostasis [24], [32], [33], we analyzed its expression in our different groups of mice. We observed higher BDNF mRNA levels in the fasting MTII injected mice than in the control fasting mice (figure 7A). Under these experimental conditions, a higher expression of COUP-TFII mRNA in the fed mice than in the fasted mice was observed, therefore the ICV *per se* did not disturb the previously observed COUP-TFII mRNA regulation by the nutritional status (figure 7C). On the other hand, the MTII-injected fasting mice displayed significantly higher COUP-TFII mRNA levels than the control fasting mice injected with CSF (figure 7C). Therefore, a central activation of the melanocortin pathway in the fasting mice induces COUP-TFII mRNA.

In fasting mice, when insulinemia and glycemia are low, an activation of the melanocortin pathway leads to an activation of COUP-TFII expression in the ventromedial hypothalamus.

### A central activation of the melanocortin pathway represses COUP-TFII mRNA levels in the liver and in the pancreas

**Figure 8:**
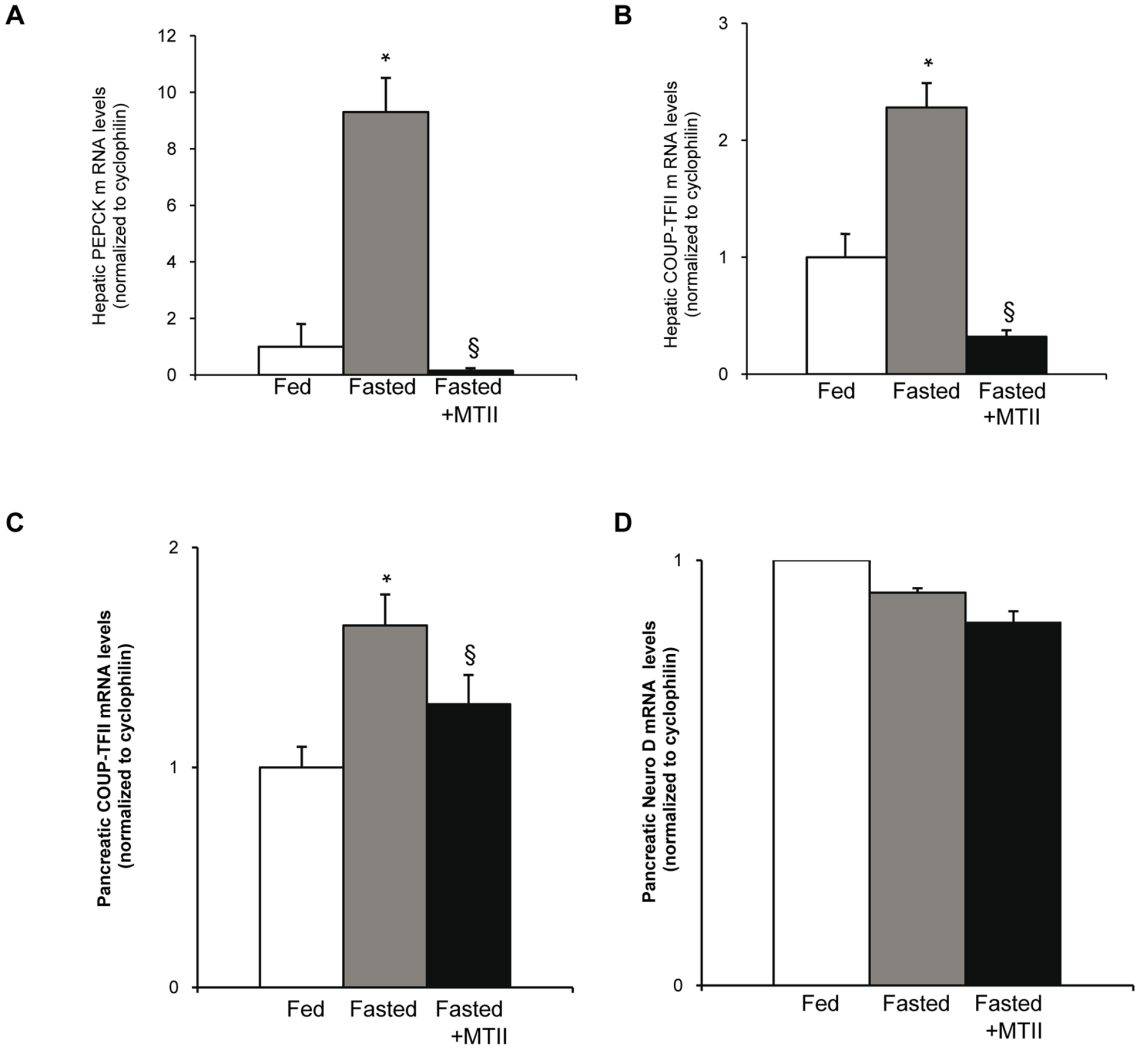
Regulation of hepatic and pancreatic COUP-TFII mRNA levels by activation of the melanocortin pathway. (A–D) RT-qPCR analysis of mRNA from livers (A–B) or pancreas (C–D) of *ad libitum* fed mice receiving a ICV injection of CSF (□), or 18 h fasting mice receiving a ICV injection of CSF (grey bar), or 18 h fasting mice with a ICV injection of MTII (▪). (A) Hepatic PEPCK mRNA levels (B) Hepatic COUP-TFII mRNA levels. (C) Pancreatic COUP-TFII mRNA levels (D) Pancreatic Neuro D mRNA levels. Data are expressed as mean ± SEM (n = 5) *P≤0.05 when compared to control fed mice receiving a CSF injection. § P≤0.05 when compared to control fasting mice receiving a CSF injection.

Previous data from our group had clearly shown that COUP-TFII mRNA levels are inhibited in the liver and in the pancreas of fed mice and by insulin in cultured hepatocytes and in a pancreatic beta cell line [7]. As an activation of the melanocortin pathway in the hypothalamus is known to affect gene expression through an activation of the autonomous nervous system, leading to variations in the phosphorylation state of the insulin signaling pathway in peripheral organs [34] [35]we asked whether a central activation of the melanocortin pathway could also contribute to the regulation of hepatic and pancreatic gene expression. In our experiments, a central MTII injection failed to lower insulin levels in fasting mice, as insulinemia was already physiologically at its lowest (figure 7D). The ICV MTII injection leads to a slight but significant increase of blood glucose concentrations (figure 7D) as previously described [36]. First, we analyzed the expression of an hepatic gene known to be modulated by a central induction of the melanocortin pathway, the phosphenolpyruvate carboxykinase gene (PEPCK), encoding an enzyme involved in hepatic gluconeogenesis. As expected, the fed state was characterized by lower hepatic PEPCK mRNA levels compared to the fasted state (figure 8A) and the central injection of MTII in fasting mice was followed by a strong inhibition of PEPCK mRNA expression, even lower than at the fed state (figure 8A). When looking at the hepatic COUP-TFII mRNA expression, it was higher in the control fasting mice compared to the control fed mice as expected (figure 8B). However, an ICV MTII injection in fasting mice strongly inhibited COUP-TFII mRNA expression in the liver (figure 8B). We also analyzed in these mouse models the pancreatic COUP-TFII expression and observed a higher expression in the control fasting mice than in the control fed mice as previously described (figure 8C) [7]. On the other hand, the MTII injection in fasting mice significantly inhibits COUP-TFII mRNA expression in the pancreas (figure 8C). The MTII injection did not alter the neuro D mRNA expression, a control gene whose expression is restricted to the pancreatic beta cells (figure 8D).

In fasting mice, a direct activation of the melanocortin pathways in the central nervous system reproduced the hypothalamic, hepatic and pancreatic gene COUP-TFII expression profiles observed in fed mice.

## Discussion

The hypothalamus is a complex structure constituted of anatomically distinct nuclei that work in a coordinate fashion to regulate metabolic functions. Among them, the ventromedian hypothalamus assumes several functions involved in sexual and defensive behaviors. In addition, experiments with lesions of the medial hypothalamus allowed the characterization of the ventromedial nucleus as the satiety center, its destruction resulting in hyperphagia and obesity [37]. More recent studies have also implicated neurons of the VMH as critical for courterregulatory responses to hypoglycemia, since sympathetic response to glucopenia are blocked by lesions of the VMH or by local infusion of glucose in the VMH [38].

In this paper, we report a COUP-TFII gene expression restricted to a subpopulation of neurons in the ventromedial hypothalamus expressing the SF1 protein and described as expressing the melanocortin receptor MC4-R [22], [23], [24], [25]. We then suggested that the fed state induction of COUP-TFII gene expression in the VMH could be indirect, through an activation of the melanocortin pathway by insulin. Indeed, even as the insulin receptors are present in neurons from the different nuclei, the highest expression is mainly described in the arcuate nuclei (ARH) [39], [40]. Moreover, binding assays with radiolabeled insulin had shown a stronger binding in the ARH compared to the VMH. These observations led to the idea that the prominent site of insulin action are the neurons of the ARH over the other hypothalamic nuclei [41]. Upon binding to its receptor in the ARH, insulin triggers a repression of the AgRP and NPY gene expression and an increase in POMC gene expression [26], [42]. The POMC gene product is αMSH which is with AgRP part of the melanocortin system. Changes in the expression of these neuropeptides in the ARH are regarded as a key mechanism mediating the insulin effect in the central nervous system. In keeping with this mechanism, we observed that in our nutritional and hormonal, eGHI and STZ mouse models, variations of circulating plasma insulin are closely associated with variation of the hypothalamic COUP-TFII mRNA levels, paralleling the modulation of POMC gene expression and therefore of the melanocortin receptor agonist (αMSH) production. Our experiments on a hypothalamic cell line with insulin showed that this hormone was able to repress COUP-TFII mRNA expression when applied directly on the cells expressing COUP-TFII as it was observed on a pancreatic beta cell line or on cultured hepatocytes [7]. *In vivo*, the observed induction of COUP-TFII gene expression by the fed state could be therefore mediated indirectly by insulin through the conjunction of decreasing amount of AgRP and increasing amount of POMC leading downstream to an activation of the MC3/4-R.

MC4-R and MC3-R have been shown to be widely distributed throughout different hypothalamic regions, with a higher expression in the ARH, the VMH, the dorso medial hypothalamus, the paraventricular nucleus and the median eminence [27], [28]. Therefore in our *in vivo* experiments with a central ventricular injection of a MC3/4-R agonist, the MTII, a complex network of neurons expressing this receptor will be activated throughout the hypothalamus. Using a hypothalamic cell line expressing the MC4-R receptor, we have shown that its direct activation induces COUP-TFII mRNA levels in these cells, so part of the MTII effect on COUP-TFII gene expression *in vivo* is mediated through a direct activation of the melanocortin receptors expressed on the VMH neurons. MC3/4-R belongs to the family of G protein-coupled seven-transmembrane-domain receptors and the binding of its agonist induces an increased cAMP production [30]. A COUP-TFII gene promoter analysis had identified cAMP response element localized in the first 300 bp from the transcription start site [31]. In our hypothalamic cell line it did drive also the cAMP dependant activation of the COUP-TFII gene. So *in vivo*, an activation of the MC3/4-R could affect COUP-TFII gene expression directly through a transcriptional mechanism regardless of the peripheral effect of MTII on the other nuclei.

Our previous studies had shown that insulin was down-regulating COUP-TFII gene expression in liver and pancreas. In regards to COUP-TFII functions in those tissues, *i.e* controlling the glucose dependant insulin secretion by the pancreatic beta cells and gluconeogenesis by the hepatocytes, this coordinated down-regulation is therefore contributing to glucose homeostasis [7]. In the hypothalamus, we observed an up regulation of its expression in fed mice, *i.e*. when peripheral insulinemia is at his highest. This apparent opposite effect of insulin on COUP-TFII gene expression is reminiscent of the overall observation regarding peripheral *vs* central insulin effects on energy homeostasis. In peripheral tissues, insulin is rightly considered as the anabolic hormone: insulin injections induce hypoglycemia with a temporary increase in food intake as a consequence and it will promote fuel storage in peripheral organs, mainly in liver and adipose tissues. On the other hand, the central effect of insulin on the hypothalamus is mainly catabolic, leading to decreased food intake and weight loss. One other striking evidence of differential regulation between hypothalamus and peripheral tissues is the fuel gauge AMPK, whose activity is decreased by leptin in the hypothalamus but increased in skeletal muscle leading to a coordinate control of fuel expenditure and food intake [43]. More recently, a protein with a crucial function in the maintenance of glucose homeostasis, the cyclic AMP-response element binding protein (CREB) regulated protein co-activator 2 (CRTC2) has been described as displaying a differential regulation by the nutritional status in liver and hypothalamus [44]. At the fed state CTRC2 is cytoplasmic in hepatocytes and nuclear in hypothalamic neurons where it will differentially regulate hypothalamic and hepatic CRE gene expression [44]. Taken all together these different observations on the peripheral *vs* central effect of the nutritional status are logical if we consider the ventromedial hypothalamus as part of the feedback loop in charge not only of the maintenance of the energy stores but also of euglycemia, a sort of check point balancing the peripheral effects of the nutritional status. Having an opposite regulation of COUP-TFII expression between these different organs could then participate in this global homeostasis.

In this paper, we described the unique and restricted localization of COUP-TFII expression to a subpopulation of VMH neurons expressing the transcription factor SF1. Actually, these VMH neurons expressing COUP-TFII are well characterized for their involvement in controlling energy homeostasis. Indeed, SF1 gene knockout mice displayed an abnormal VMH development leading to obesity [23]. This demonstrates, outside of SF1 function in neuronal development, the involvement of such neurons in the control of food intake and fuel expenditure [23]. These neurons are known to control food intake through the production of BDNF, a neurotrophin whose expression in the VMH is increased in response to the activation of MC3/4-R [24], [32], [33].

These COUP-TFII positive neurons also express the MC4-R. Since mutations in the human MC4-R or deletion in mouse models have been associated with obesity, this receptor has received much attention as a potential drug target to treat this pathology [45], [46], [47], [48]. Interestingly, most of this attention has been brought on its involvement in the control of food intake but it has been clearly shown that the melanocortin pathway play a global role in energy homeostasis. It stimulates oxygen consumption, inhibits basal insulin release and glucose tolerance, and this impaired insulin intolerance occurs prior to the onset of hyperphagia and obesity [49]. These data suggest that the central melanocortin system regulates not only energy intake, but also energy partitioning, as indicated by effects on insulin release and peripheral insulin responsiveness. As much as we know of the natural agonist and antagonist of this receptor, αMSH and AgRP, there is a lack of identified downstream effectors of this receptor. Indeed, so far, very few targets have been clearly identified. In the VMH and the dorsomedial hypothalamus, the BDNF gene expression is clearly modulated by MC4-R activation and several lines of evidence have also identified the Single Minded 1 as a downstream target of MC4-R in the paraventricullar hypothalamus [50], [51]. Identifying downstream target genes, such as COUP-TFII, could be the first step in understanding the molecular mechanisms underlying the melanocortin-mediated regulation of energy homeostasis. Undoubtedly, mice models with a specific invalidation of its expression restricted to the VMH neurons will be an invaluable tool in deciphering the proper implication of COUP-TFII as a central component of the hypothalamic integration of energy status and energy balance regulation.

In conclusion, COUP-TFII expression is regulated in the VMH by insulin levels aside of circulating glucose and this suggests a possible role as a modulator of the counter regulation engaged by the VMH in response to neuroglucopenia or hypoglycemia.

## Materials and Methods

### Animals

C57bl/6J male mice (Charles River, France) were used in this study. They were adapted to the facility environment for 2 weeks prior to the different studies. Animals were housed in colony cages with a 12-h light/12-h dark cycle in a temperature-controlled environment and fed *ad libitum* with a standard laboratory chow diet: composition 65% carbohydrate, 11% lipids, and 24% proteins (SAFE, France). All animal protocols were undertaken according to the Guidelines for Care and Use of Experimental Animals. Animal experimentation permit number 75-739 (Certificat d'autorisation d'expérimenter sur animaux vertébrés number 75-739) given by the veterinarian services board (Dr. Claudine Crochet, D.V.M). Animal care facility agreement number A-75-14-02.

### Streptozotocin injections

Ten weeks old C57bl/6J male mice were used in these experiments. Streptozotocin (STZ) is dissolved extemporaneously in citrate buffer at pH 4.5 and administered daily at 180 mg/kg body weight (Sigma, St. Louis, MO, USA) for two days. Control mice received two citrate buffer injections. Food and water were provided *ad libitum* during the remaining 7 days of the experiment. After the injection, mice were checked daily for plasma glucose concentration *via* the tail vein with a blood glucometer (Roche diagnostic, Meylan, France). A subgroup of STZ treated mice received an intraperitoneal injection of insulin (1.5 U/kg) (Actrapid, Novo Nordisk) 2 hours prior to the sacrifice.

### Euglycemic-hyperinsulinemic (eGHI) clamped mice

Ten weeks old C57bl/6J male mice were used in the eGHI experiments. Mice were catheterized at least 2 days before the experiment. They were anesthetized with a mixture of ketamine (100 mg/kg of body weight) and xylazine (10 mg/kg of body weight). The right jugular vein was catheterized with a silastic catheter for infusion. The free end of the catheter was tunneled under the skin to the back of the neck and passed through a piece of Tygon tubing, glued together, and secured to the skin [7]. Lines were flushed daily with 50 µl of 0.9% NaCl containing 5 mg/ml ampicillin and 20 IU/ml heparin. After surgery, animals were housed individually and weighed daily. After a 5 h fast on the day of experiment, awaken animals were placed unrestrained into their cage during the perfusion. Two groups of mice were studied: a saline-infused (control group) and glucose infused/insulin-infused (euglycemic-hyperinsulinemic [eGHI]) group. In eGHI mice, hyperinsulinemia was induced by a 3-h insulin infusion at a constant rate of 0.6 IU/kg/h (Actrapid; Novo-Nordisk) and euglycemia was maintained by a simultaneous glucose infusion at a flow rate adjusted to the basal glycemia, around 100 mg/dl. Blood glucose levels were determined from tail blood samples at time zero and then every 15 min. Steady state was reached when glucose measurements were constant for at least 20 min at a fixed glucose infusion rate and was achieved within 30 to 45 min. Plasma insulin concentrations were measured with a radioimmunoassay kit (Insik-5 kit; Diasorin). At the end of the infusion, mice were killed by pentobarbital injection, and the hypothalamus frozen in liquid nitrogen and kept at −80°C until analysis.

### Cell culture

The mouse GT1-7 hypothalamic cell line [52] was used between passage 31 and 37. The cells were grown in 5% CO2-95% air at 37°C in Dubelco modified Eagle medium (DMEM) containing Glutamax 1x supplemented with 10% (vol/vol) of heat inactivated fetal calf serum and 100 U/ml penicillin-streptomycin. For melanotan II (MTII) (Phoenix pharmaceuticals, Burlingame, CA, USA) or Dibutyryl-cAMP (DiBcAMP) (Sigma, St. Louis, MO, USA) dose response experiments, cells were seeded in 12 well plates at a density of 150,000 cells/well. Twenty four hours after plating, cells were cultured in presence of MTII or DiBcAMP for 16 h prior to the RNA extraction.

### Plasmids, transfection and reporter gene assay

The reporter plasmid -328 (Apa I) to +873 (BglII) relative to the transcription initiation site was described in Perilhou *et al*. (-328/luc) [53]. Cells were plated at density 150,000/well in GT1-7 medium. Transient transfections were carried out with Lipofectamine 2000 reagent (Invitrogen, Cergy, France), 800 ng of reporter plasmid and 100 ng of internal control. The following day the cells were treated with DiBcAMP for 6 hours and cell extracts were assayed for reporter enzyme activities, using the dual luciferase kit (Promega, Charbonnieres, France) as described previously [53].

### Isolation of total mRNA and quantitative analysis by real time RT-PCR

Total RNAs were extracted and purified from hypothalamus, cultured GT1-7 cells and liver using the RNA-Plus reagent (Q-BIOgene, Montreuil, France) according to the manufacturer instructions. For RNA extraction from flash frozen pancreas, the tissues were powdered in liquid nitrogen using a steel mortar and pestle. A frozen minute amount of powdered pancreas was then immediately homogenized in a chaotropic buffer and RNA were extracted using the RNAeasy kit (Qiagen, Courtaboeuf, France) according to the manufacturer instructions. Reverse transcription was performed with 1 µg of total RNA using the Superscript II reverse transcriptase (Invitrogen, Cergy, France). Real time PCR was performed using 6.25 ng of reverse-transcribed RNA subsequently mixed with Fast Start DNA Master Sybr Green (Roche, Meylan, France), 2 mM MgCl2 and various set of gene specific forward and reverse primers in a final volume of 10 µl. The relative amount of each mRNA was calculated using the 2^nd^ derivative Ct method. Quantification was obtained after normalization to cyclophilin mRNA and inter-assay variations using an RT-calibrator [54].

Forward and reverse primers used for the specific amplification of mouse cDNA fragments were: COUP-TFII: 5′-CGC TCC TTG CCG CTG CT-3′ and 5′-AAG AGC TTT CCG AAC CGT GTT-3′, Cyclophilin: 5′-TTG CCA TTC CTG GAC CCA AA-3′ and 5′-ATG GCA CTG GTG GCA AGT CC-3′ Neuro D1: 5′-GCC CAG CTT AAT GCC ATC TTT-3′ and 5′-CAA AAG GGCTGC CTT CTG TAA-3′ Brain-derived neurotrophic factor (BDNF): 5′-CAG GGG CAT AGA CAA AAG -3′and 5′-CTT CCC CTT TTA ATG GTC-3′, Pro-opiomelanocortin (POMC): 5′-ATG CCG AGA TTC TGC TAC AGT CG-3′and 5′-TC ATC TCC GTT GCC AGG AAA CAC -3′, Agouti related protein (AgRP): 5′-TT TGG GAG AGC GTG TAA CTG AA-3′and 5′-CT CCA TAT CGT GGT CTG AAA CTG-3′, Phosphoenolpyruvate carboxykinase (PEPCK): 5′ GTG CTG GAG TGG ATG TTC GG 3′and 5′ CTG GCT GAT TCT CTG TTT CAG G 3′, Neuropeptide Y(NPY): 5′ TTC TGT GCT TTC CTT CAT 3′ and 5′ GTG TTT GGG CAT TCT G 3′.

### Western blot analysis and ECL detection

Nuclear extracts were prepared from fresh tissues as described previously by Bossard *et al*. [55]. Western blot were done using 30 µg of nuclear extracts and according to Perilhou *et al*. [7].

### Immunohistological studies

Two-month-old C57Bl/6J male mice were decapitated and their brains were removed, frozen at −35°C for 2 minutes in isopentane and stored at −80°C. Frozen brains were mounted and placed in a cryostat and 7 µm sections were cut in the coronal plane. One section out of 20 was stained by the Kluver Berrera method which by staining the myelin in a bluish hue and the cells structures in violet allows the visualization of the different brain structures [56]. For COUP-TF immunostaining, sections were fixed in 1% paraformaldehyde for 20 min at 4°C then permeabilized with 0.1% triton in 1X Phosphate buffer saline (PBS) for 10 min at room temperature. The sections were incubated with a mouse IgG blocking agent (Mouse-on-Mouse, Vector, USA), then incubated overnight at 4°C with mouse monoclonal primary antibodies: COUP-TFII/NR2F2 (PP-H7147-00, dilution 1∶50) or the COUP-TFI/NR2F1 (PP-H8132-00, dilution 1∶100). The following day, the sections were washed and revealed using an anti-mouse antibody coupled with a peroxidase labeling enhancement system (Envision DAB-HRP, Dakocytomation). On the same slide, a serial section was treated without the primary antibody as a control for secondary antibody specificity. For immunofluorescence double staining, the slides were incubated overnight at 4°C with mixed primary antibodies directed against SF1 (rabbit anti SF1 polyclonal antibody, Ab3436, AbCam, dilution 1∶100) and against COUP-TFII (PP-H7147-00, dilution 1∶50), or against COUP-TFII and MC4-R (goat anti MC4-R polyclonal antibody, R-19, Santa Cruz, dilution 1∶100), then with mixed secondary antibodies: FITC conjugated anti-mouse and Texas Red conjugated anti rabbit for SF1/COUP-TFII double staining or Alexa 568 conjugated anti-mouse and Alexa 488 conjugated anti-goat for MC4-R/COUP-TFII double staining, at room temperature for 1 h. The slides were mounted in DAPI (4-6-diamidino-2-phenylindole) containing Vectashield (Vector Laboratories, Philadelphia, PA, USA) for the nuclei counterstaining and stored at 4°C until observation. The different specificity controls were done on the same slides: a section was incubated with only one of the primary antibody then incubated with the mixed secondary antibodies. A serial section is also treated without any primary antibody, as a control for the secondary antibodies specificity.

### Intracerebroventricular (ICV) injection

Twelve weeks old C57bl/6J male mice were anaesthetized with isofluran (2.5%). After the initial anaesthesia, they were connected at the snout to a device providing isofluran at 1.5% for the surgery duration. As soon the animals were anaesthetized, they received an injection of xylazine and xylocaine (10 mg/kg of body weight). A chronic guide cannula (26 ga, Plastic One Products, Roanoke, VA) was implanted into the right lateral brain ventricle to coordinates X = −1 mm, Y = −0.4 mm, Z = −2.5 mm from the Bregma using a Kopf stereotaxic instrument (David Kopf Instruments, Tujunga, CA, USA). The 26 stainless steel gauge guide cannula (Plastic One Products, Roanoke, VA, USA) was maintained in place by dental cement anchored to one stainless steel jewelry screw fixed to the skull. A 28 gauge dummy cannula was inserted to prevent clogging of the guide cannula. After the surgery, the animals were housed individually with direct bedding for 10 days during which they were handled (about 5 min daily) and accustomed to the placement of the ICV injector. Paracetamol was added to the drinking water for the first 2 days. Body weight was accessed daily. For the ICV injection, an injector was connected to a catheter containing the MC4-R agonist MTII or its vehicle (artificial cerebrospinal fluid (CSF) (Alzet, Cupertino CA, USA)) and inserted into the guide cannula in the non restrained conscious mice. This catheter was connected to a Hamilton syringe powered by a mini-pump KDS310 (KD Scientific, Holliston,MA, USA).

A small air bubble (1 µl) was drawn at the distal end of the catheter to separate the injected solution from the water and for visual inspection of injections which were performed slowly over a 50–60 sec period. The volume was 1 µl in a single injection at the rate of 1 µl/min. Only mice who recovered their normal body weight one week after the surgery were included in this study.

### Statistical analysis

Quantitative results are expressed as means ± standard errors of the means (SEM). Statistical analyses are carried out using the Mann-Whitney test, a nonparametric statistical program appropriate for sample number less than 10. Null hypotheses are rejected at *P* values of ≤0.05.
